# Gradual ulnar lengthening in children with multiple exostoses and radial head dislocation: results at skeletal maturity

**DOI:** 10.1007/s11832-016-0718-8

**Published:** 2016-02-24

**Authors:** Riccardo D’Ambrosi, Alessia Barbato, Camilla Caldarini, Elena Biancardi, Renato Mario Facchini

**Affiliations:** Università degli Studi di Milano, Milan, Italy; Centro Traumatologico Ortopedico, U.O. Clinica Ortopedica e Traumatologica, Milan, Italy

**Keywords:** Forearm deformities, Radial head dislocation, Hereditary multiple exostoses, Ulnar lengthening, External fixation, Skeletal maturity

## Abstract

**Purpose:**

Deformities of the forearm and shortening of the ulna occur in 30 % of patients with hereditary multiple exostoses (HME), leading to radial head dislocation and loss of movement. Several surgical techniques have been described for treatment, and the aim of our study was to present clinical and radiographic results at skeletal maturity in 15 children that underwent the surgical procedure of ulnar lengthening with external fixators.

**Methods:**

We evaluated 15 patients with ulnar shortening and radial head dislocation that underwent external fixation procedures. Radiographic assessment included measurement of radial articular angle, carpal slip, and ulnar shortening. Clinical evaluation included range of motion, MAYO Elbow Score, assessment function of the extremity as described by Stanton, the visual analog scale (VAS) for pain, and SF-12 to evaluate quality of life.

**Results:**

The average follow-up period was 77 months and took place when each patient had reached skeletal maturity. MAYO Elbow Score improved from 34.7 to 93.3 points, while the average preoperative functional assessment criteria score was 1.6 points and improved to 4.4. The preoperative average VAS ranged from 8.2 to 2.3, while the SF-12 in its physical (PCS) and mental (MCS) components resulted, respectively, as 53.3 and 54.2. Pronation and supination improved from a preoperative average value of 35.6° and 51.3° to 70° and 80.6°, respectively, at the most recent follow-up visit. Flexion and extension ranged, respectively, from 143° and 2° to 146.7° and 3°. Ulnar shortening improved from 24 mm preoperative to 3 mm, and radial articular angle varied from 37.7° preoperative to 26° at the last follow-up. Only one complication occurred in our group, and one patient completely healed from a case of nonunion of the ulna.

**Conclusions:**

Ulnar lengthening is a safe and reliable procedure for the treatment of HME that provides good to excellent results and reduces radial head dislocation.

## Introduction

Deformities of the forearm occur in 30 % of patients with hereditary multiple exostoses (HME) [1, 2]. Osteochondromas can cause several complications such as deformities, fractures, and impingements, although malignant transformation is rare [3, 4]. The involvement of the upper limb is associated with radial head dislocation that leads to reduction of forearm rotation and functional impairment, altering the normal activities of daily living [1, 5]. Shortening of the ulna associated with radial head dislocation are common conditions in patients with HME, and surgical treatment should always be taken into account if complications occur, such as pathological fractures, vascular and nerve damage, synovial cysts, infection, and malignant transformation [6–8]. Moreover, if the course of the disease is long, adaptive pathological changes such as radial head deformation or a bow-shaped deformity caused by radial overgrowth may occur secondary to chronic joint dislocation [9]. Biomechanical studies have shown that ulnar shortening occurs because the distal physis contributes to the total length of the radius more than the distal radial physis does [10–12]. Moreover, the distal ulnar physis has a smaller cross-sectional area than the radial one, causing a greater proportional involvement of the ulnar growth plate; differential longitudinal growth on the radial and ulnar border results in an increase in the distal radial articular angle, and ultimately in radial head dislocation [13]. Several surgical techniques have been described for the treatment of this kind of deformity [12, 14–17]. Pain or functional deficit related to an osteochondroma are indications that surgery is required. Functional deficit means the loss of motion needed to perform 90 % of day to day activities, which equals to 30° for extension, 130° for flexion and 50° both for pronation and supination [18, 19]. Surgical procedures include simple excision of osteochondromas, radial head osteotomy, acute or gradual ulnar lengthening, corrective radial osteotomy, hemi-epiphyseal stapling of the distal radius, creation of a one-bone forearm, and the Sauvé-Kapandji procedure.

For years, simple excision and radial head osteotomy have been considered the gold standard for the treatment of forearm exostoses, but only minimal improvement in the rotation of the forearm was noted by sacrificing the radial head [12, 14, 20]. Ulnar lengthening diminishes ulnar support for the carpus and increases ulnar-sided pressure on the radial epiphysis. With this technique it is possible to restore the axis of the forearm, reduce radial head dislocation, and recover the movements of pronation and supination [21–24]. Because of its effectiveness and excellent results, this technique is becoming increasingly popular and is being used by surgeons dealing with pediatric orthopedics, although the results are not widely documented in the literature. The aim of our study was to examine the clinical and radiographic results at maturity in 15 children that underwent surgical ulnar lengthening with external fixators to treat radial head dislocation and ulnar shortening.

## Material and methods

We retrospectively reviewed 15 patients suffering from HME with osteochondroma-induced forearm deformities and radial head dislocation that underwent fixator-controlled correction procedures between 2004 and 2008.

Indications for surgery included that the patients had not reached skeletal maturity, had worsening of symptoms and deformity causing disturbance in daily activities, ulnar shortening (US) of 15 mm or more or exceeding 8 % [25], radial head dislocation associated with severe and chronic pain (VAS ≥ 7), and loss of function. For loss of function we evaluated a movement of extension inferior to 30°, flexion inferior to 130°, and pronation or supination inferior to 50° [19], or a loss of function regarding pronation or supination superior to 40 % as compared with the contralateral. None of the patients presented with bilateral involvement.

The patients underwent standardized clinical and radiologic evaluations twice: preoperatively and once skeletal maturity had been reached. All surgical procedures were performed by the senior author.

**Fig. 1:**
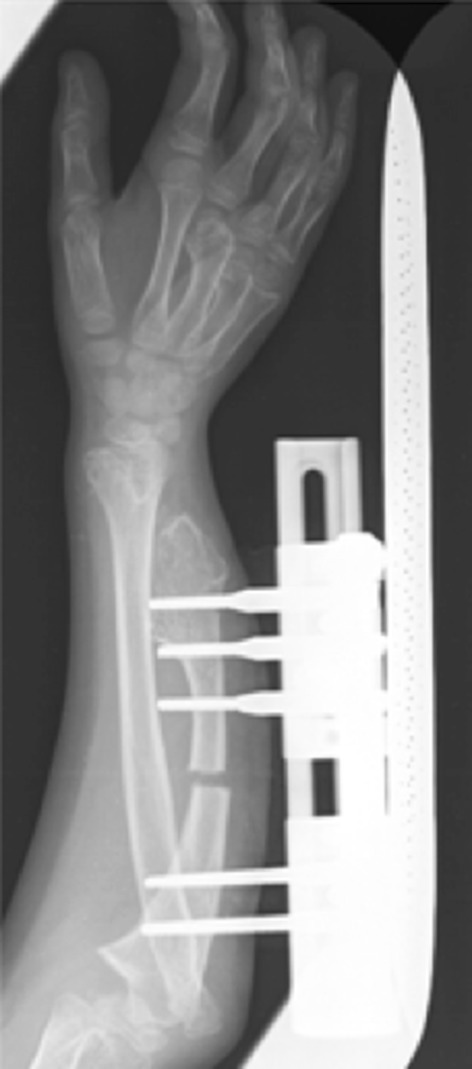
Anteroposterior radiograph of a forearm 15 days after surgery: Correct positioning of the external fixator, removal of exostosis, and osteotomy of the ulnar shaft

The patients were operated on in the supine position under general anesthesia with the forearm on a radiolucent table. Before surgery, the forearm was maintained in a neutral position. The operative technique consisted of the removal of all the osteochondromas present in the ulna and the reduction of radial head dislocation. If the patient presented with a radio-ulnar distal synostosis, a radioulnar separation was performed. Subsequently, an ulnar transverse mid-diaphyseal subperiosteal osteotomy was performed and a unilateral external fixator was positioned. Before starting with the progressive lengthening we waited 4–7 days to let the hematoma appear, which stimulates callus formation. Once the elongation was obtained, the fixator was maintained for the time needed to reach the desired length. Care was taken to avoid neurovascular injury, swelling, and pin tract infection (Fig. 1). The external fixator was removed after an average of 135 (range 120–150) days.

Radiological assessment consisted of standard forearm antero-posterior and lateral radiograph in an anatomic position. Forearm deformity was classified according to Masada [14].

**Fig. 2:**
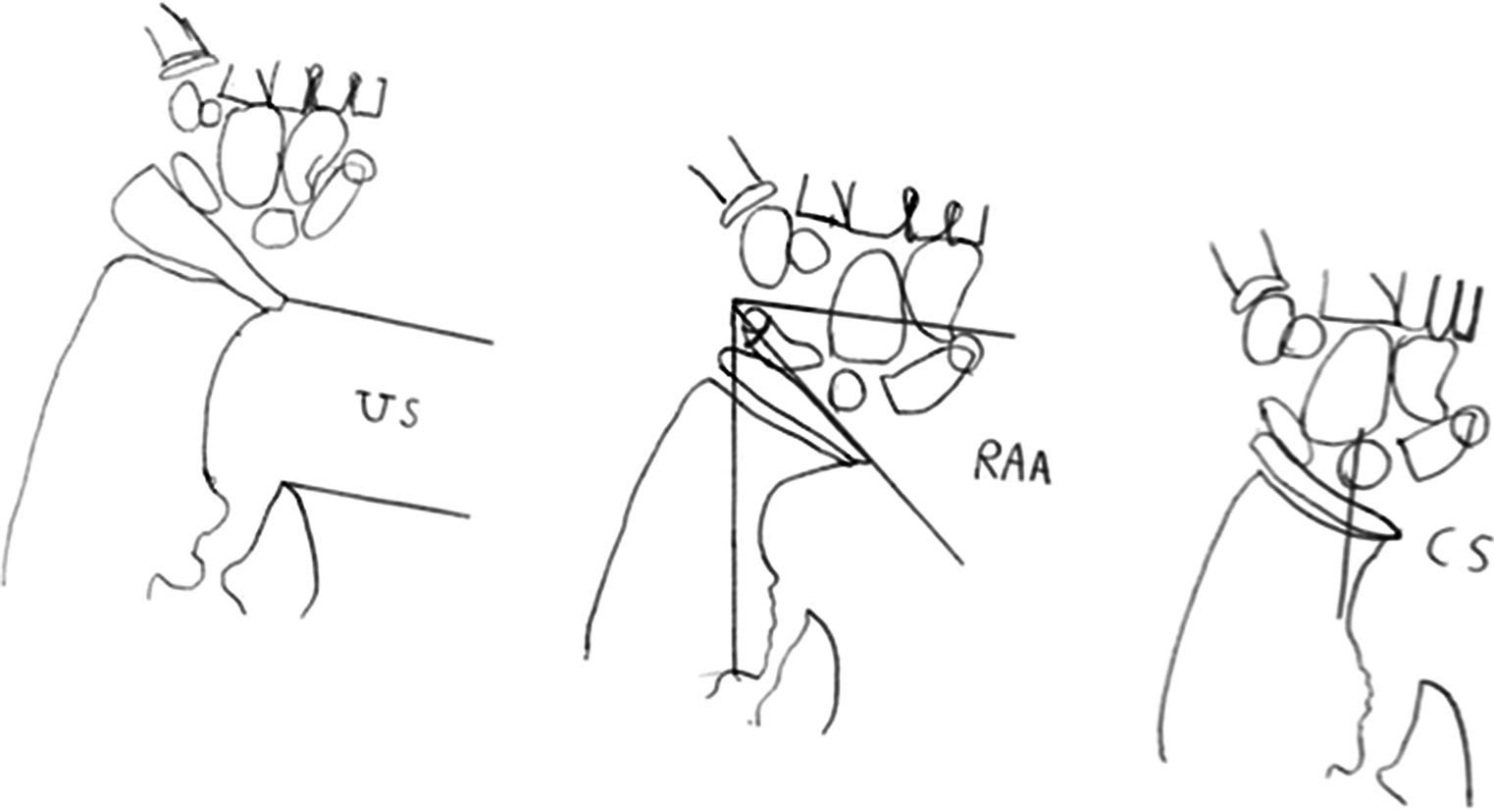
**a** *US* is measured with the perpendicular line drawn from the distal end of the ulna to the linear axis of the forearm; **b** *RAA* is the angle between a line drawn along the articular surface of the radius and the other perpendicular to a line that bisects the head of the radius and passes through the radial edge of the distal radial epiphysis; **c** *CS* is measured as the percentage of contact of the lunate with the radius. This percentage is determined by drawing an axial line from the center of the olecranon through the ulnar edge of the radius

Radiographic assessment included radial articular angle (RAA), carpal slip (CS), ulnar shortening and radial head dislocation (Fig. 2). Relative US, RAA, and CS were measured according to the method described by Fogel [12]. We measured the amount of ulnar lengthening (AUL) at the most recent follow-up and calculated the external fixation index (EFI). EFI was obtained by dividing the total duration of external fixation by the length gained.

Clinical evaluation included the range of motion of the elbow at the preoperative visit and at the most recent visit to the outpatient department and MAYO Elbow Score [26]. Moreover, each patient was asked to rate the function of the extremity according to specific criteria as described by Stanton and the SF-12 to assess their own quality of life [5, 27] (Table 1).

**Table 1:** Functional assessment criteria by the patient

5	I have no limitations of my activities and no pain
4	I have no pain. I have some limitation of my activities but have not had to change my life (sports activities or job) because of it
3	I have no pain. I have had to change or limit my job or give up certain sports activities because of the condition of my hand
2	I have pain in my hand, wrist, or elbow, but I have no limitations because of it
1	I have pain in my hand, wrist, or elbow, which limits my activities
0	I have pain for which I take medications

The statistical analysis was performed using dedicated statistical software (SPSS version 17, SPSS Inc., IBM, Chicago, IL, US). Continuous variables were reported as ranges. For continuous variables a paired-samples t-test was performed to compare pre- and post-operative values. For all the tests, *p* values <0.05 were considered statistically significant.

All radiological measurements were made using the standard tools in our picture archiving and communication system (PACS) and evaluated by two orthopaedic surgeons not involved in the surgical procedures.

## Results

**Fig. 3:**
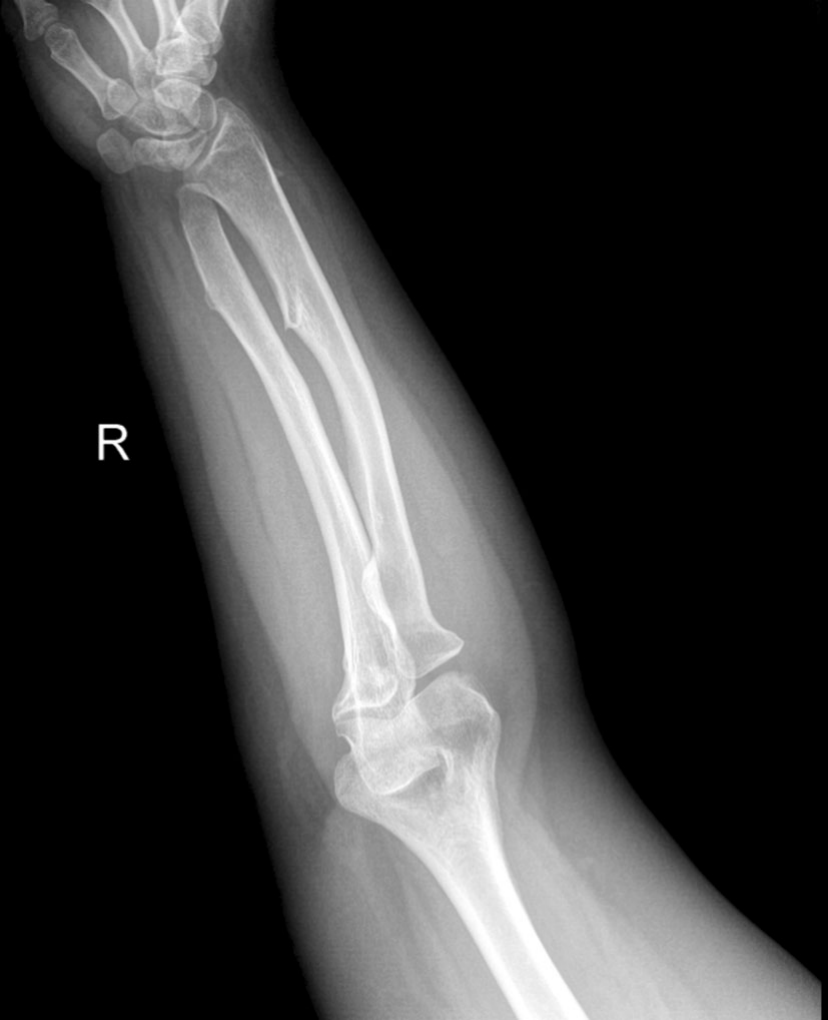
Anteroposterior radiograph of a forearm at skeletal maturity: the radial head appears in the correct position

No patients were lost at the final follow-up stage. The average age at surgery was 10.1 years (range 8–12). Surgery involved seven (46.7 %) left forearms and eight (53.3 %) right forearms in seven (46.7 %) females and eight (53.3 %) males. The average age at follow-up stage was 18.2 years (range 17–23) and the average follow-up period was 77 (range 50–95) months after surgery, when every patient had reached skeletal maturity (Fig. 3). We noted six (40 %) Type IIA deformities, eight (53.3 %) Type IIB, and one (6.7 %) Type III according to the Masada classification (Table 2).

**Table 2:** Demographic data

Age	10.1 (8–12)
Sex	
Male	8
Female	7
Side	
Right	8
Left	7
Follow-up (months)	77 (50–95)
Masada grade	6 IIA, 8 IIB, 1 III

The average preoperative MAYO Elbow Score was 34.7 points (range 15–50) and had improved significantly to 93.3 (range 85–100) at the final follow-up (*p* < 0.05). The average preoperative patient score was 1.6 (range 0–2) points and improved significantly to 4.4 (range 3–5) (*p* < 0.05). Visual analog scale (VAS) scores improved significantly from an average preoperative of 8.2 (range 7–9) to 2.3 (range 1–4) (*p* < 0.05). Scores from the SF-12 at skeletal maturity were, respectively, 53.3 (range 43.3–55.9) and 54.2 (range 41.8–58.8) for the physical (PCS) and mental (MCS) components.

Pronation improved from a preoperative average of 35.6° (range 0°–80°) to 70° (range 45°–90°) at the latest follow-up (*p* < 0.05); movement of supination improved from a preoperative average of 51.3° (range 0°–90°), to 80.6° (range 60°–90°) at the latest follow-up (*p* < 0.05).

Flexion improved from 143° (range 130°–160°) to 146.7° (range 130°–160°) at the final follow-up, while movement of extension ranged from 2° (range 0°–5°) preoperatively to 3° (range 0°–5°) (*p* < 0.05) (Table 3).

**Table 3:** Clinical results at skeletal maturity

	Preoperative (range)	FFU (range)	*p*
MAYO Elbow Score	34.7 (15–50)	93.3 (85–100)	<0.05
Functional assessment criteria	1.6 (0–2) (1 grade 0, 5 grade 1, 8 grade 2, 1 grade 3)	4.4 (3–5) (1 grade 3, 7 grade 4, 7 grade 5)	<0.05
Pronation	35.6° (0°–80°)	70° (45°–90°)	<0.05
Supination	51.3° (0°–90°)	80.6° (60°–90°)	<0.05
Flexion	143° (130°–160°)	146.7° (130°–160°)	ns
Extension	2° (0°–5°)	3° (0°–5°)	<0.05
VAS	8.2 (7–9)	2.3 (1–4)	<0.05
SF-12			
MCS	na	53.3 (43.3–55.9)	na
PCS	na	54.2(41.8–58.8)	na

*p* values <0.05 Statistically significant

*FFU* final follow-up, *ns* non significant, *na* not available

Radiological assessment also showed significant improvement: US had improved from 24 mm preoperative (range 15–44) to 3 mm (range 0–8) (*p* < 0.05); RAA had varied from 37.7° preoperative (range 28°–49°) to 26° (range 22°–31°) at the most recent follow-up (*p* < 0.05) (Table 4).

**Table 4:** Radiographic results at skeletal maturity

	Preoperative	FFU	*p*
Ulnar shortening (mm)	24 (15–44)	3 (0–8)	<0.05
Radial articular angle	37.7° (28°–49°)	26° (22°–31°)	<0.05

*p* values <0.05 Statistically significant

*FFU* final follow-up

Carpal slide was positive in seven (46.7 %) patients before surgery, and remained positive in three (20 %) patients at final follow-up. No cases of dislocation were reported.

The average AUL and duration of external fixation index were 24.5 (range 15–42) mm and 134.9 (range 120–150) days, respectively. Thus, the external fixation index was an average of 5.8 (range 3.4–7.8) day/mm.

In three (20 %) patients the presence of radio-ulnar distal coalition required radioulnar separation and removal of exostoses simultaneously to the lengthening of the ulna.

**Fig. 4:**
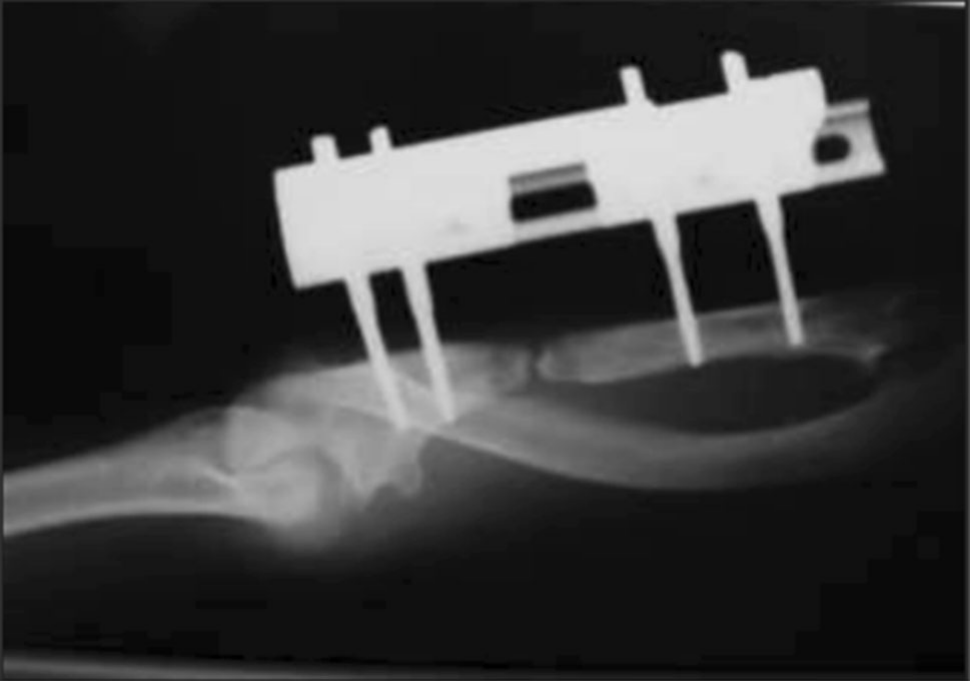
Anteroposterior radiograph of the forearm: nonunion of the ulna

Only one complication occurred in our patient group. One patient presented a case of nonunion of the ulna (Fig. 4). This was resolved by removing the external fixator and proceeding to bone cruentation; a bone block harvested from autologous iliac crest was then inserted, and was synthesized with a locking compression plate (LCP) plate.

## Discussion

The aim of our study was to report the outcomes of 15 patients who underwent progressive ulnar lengthening for radial head dislocation due to exostoses of the ulna. All patients were evaluated once they reached skeletal maturity.

The average MAYO Elbow Score was 34.7 points and improved significantly to 93.3; the average preoperative functional assessment score ranged from 1.6 to 4.4; VAS improved significantly from an average preoperative value of 8.2 to 2.3 at the final follow-up. Patients reported high values even with regard to quality of life; in fact, the SF-12 scores for physical and mental components were, respectively, 53.3 and 54.2.

Pronation and supination improved, respectively, from a preoperative average of 35.6° and 51.3° to 70° and 80.6° at the latest follow-up; flexion and extension ranged, respectively from an average of 143° and 2° to 146.7° and 3° at skeletal maturity. Radiological assessment showed improvement in all the variables considered: US improved from 24 to 3 mm, while RAA varied from 37.7° preoperatively to 26°. These results show how a significant reduction in range of motion of the elbow, as reported by Morrey [19], can greatly affect daily activities and hence, the patient’s quality of life.

To our knowledge, our study includes the greatest number of patients and the longest follow-up period compared to any other study.

No patient reported radial head dislocation. It is important to note that our group was homogenous in regards to age and type of pathology. In addition, the follow-up visit in all patients was performed after skeletal maturity had been reached, showing how this technique can be considered safe and reliable over time.

We decided to perform this technique on patients because the rationale for ulnar lengthening is that the hypoplastic ulna, the keystone of the complex deformity, tethers the radial physis, theoretically diminishing ulnar support of the carpus and increasing ulnar-sided pressure on the radial epiphysis [12, 14, 15].

In recent years, acute or gradual lengthening of the ulna has become an increasingly popular technique and several studies in medical literature report on the results of this technique, but with conflicting results; Fogel, for the first time in 1984, performed ten ulnar lengthening procedures with no significant improvement in rotation of the forearm, radial articular angle, or carpal slip [12].

Subsequently, Pritchett reported the results of ulnar lengthening in ten forearms. Lengthening was performed by osteotomy of the shaft followed immediately by a bone graft and gradual distraction with an external fixator. In all patients the appearance was improved and the range of radial deviation at the wrist was increased. In most patients, forearm movement and radial head stability were improved [15].

Masada confirms the good results achieved using this technique, achieving 92 % of satisfactory results in 16 forearms [14]. Masada also describes a treatment algorithm for the deformities related to exostoses of the forearm: excision of osteochondromas, immediate ulnar lengthening, and corrective osteotomy of the radius for Type I deformity; excision of the radial head is necessary for Type IIA, gradual lengthening of the ulna using an external fixator for Type IIB, and excision of osteochondromas alone in Type III deformities.

In 2006, Matsubara treated seven forearms by excision of osteochondromas, correction of radii, and gradual lengthening of ulnas with external fixators, achieving satisfactory results, especially for function of the elbow and wrist. However, the author cited the possible recurrence of ulnar shortening within about 1.5 years during skeletal growth periods in immature patients [23].

In contrast, Akita showed no beneficial results with ulnar lengthening in patients with multiple exostoses, affirming that simple exostoses excision is reasonable when forearm pronation is restricted [22].

Similar results were obtained from Shin, who reported no statistically significant clinical or radiological improvements in his group of patients [24].

Over the years different techniques have been described for the treatment of deformities of the forearm, in particular caused by HME, but without effective and lasting results over time [14–17].

Simple excision of the exostoses and radial head osteotomy have been considered the gold standard for years, but results reported in literature are conflicting: simple excision leads to an improvement in range of motion but will not halt the progression of the disease [12–15, 24].

Many authors consider the Sauvé-Kapandji procedure in conjunction with simple excision of osteochondromas a useful method for treating deformity of the forearm in patients with multiple hereditary osteochondromas: this leads to improvement of the stability of the wrist, movement of the forearm, and radiological appearance. In many cases, however, an additional surgical procedure is required [24, 28].

In our group of patients we decided to intervene before they reached skeletal maturity; the ideal time for surgery, however, remains an open debate. In fact, several authors suggested early intervention, due to greater potential for remodeling, which in turn would lead to better surgical results; however, recurrence of ulnar shortening was noted following the lengthening procedure [10, 14].

Intervention at a later age can be recommended because a recurrent operation can be avoided by postponing the procedure, and good function can be acquired despite significant deformity after skeletal maturity [20, 29, 30].

Our study presents some limitations. Despite a long-term follow-up period and a relatively high number of patients as compared with other studies, there was no control group.

## Conclusion

The scale of the patient group and the length of the follow-up period of this study is unprecedented in literature. This study represents the longest follow-up period and the conclusion that ulnar lengthening is a safe and reliable procedure for the treatment of HME, providing good to excellent results in the long term. Clinic and radiographic long-term follow up confirm the excellent results of ulnar lengthening with external fixation.
